# Rational truncation of aptamer for cross-species application to detect krait envenomation

**DOI:** 10.1038/s41598-018-35985-1

**Published:** 2018-12-12

**Authors:** Abhijeet Dhiman, Anjali Anand, Anita Malhotra, Eshan Khan, Vishal Santra, Amit Kumar, Tarun Kumar Sharma

**Affiliations:** 1 Department of Biotechnology, All India Institute of Medical Sciences (AIIMS), New Delhi, 110029 India; 2 Centre for Biodesign and Diagnostics, Translational Health Science and Technology Institute (THSTI), Faridabad, 121001 Haryana India; 3 School of Biological Sciences, College of Natural Sciences, Bangor University, Bangor, LL57 2UW UK; 4 Discipline of Biosciences and Biomedical Engineering, Indian Institute of Technology Indore, Simrol, Indore, 453552 India; 5 Simultala Conservationists (Foundation for Wildlife), Nalikul, Hooghly, 712407 West Bengal India; 6 Faculty of Pharmacy, Uttarakhand Technical University (UTU), Dehradun, 248007 Uttarakhand India

## Abstract

In majority of snakebite cases, the snake responsible for the bite remains unidentified. The traditional snakebite diagnostics method relies upon clinical symptoms and blood coagulation assays that do not provide accurate diagnosis which is important for epidemiological as well as diagnostics point of view. On the other hand, high batch-to-batch variations in antibody performance limit its application for diagnostic assays. In recent years, nucleic acid aptamers have emerged as a strong chemical rival of antibodies due to several obvious advantages, including but not limited to *in vitro* generation, synthetic nature, ease of functionalization, high stability and adaptability to various diagnostic formats. In the current study, we have rationally truncated an aptamer developed for α-Toxin of *Bungarus multicinctus* and demonstrated its utility for the detection of venom of *Bungarus caeruleus*. The truncated aptamer α-Tox-T2 (26mer) is found to have greater affinity than its 40-mer parent counterpart α-Tox-FL. The truncated aptamers are characterized and compared with parent aptamer for their binding, selectivity, affinity, alteration in secondary structure and limit of detection. Altogether, our findings establish the cross-species application of a DNA aptamer generated for α-Toxin of *Bungarus multicinctus* (a snake found in Taiwan and China) for the reliable detection of venom of *Bungarus caeruleus* (a snake found in the Indian subcontinent).

## Introduction

Snakebite is one of the major neglected public health problems that primarily affect rural populations in tropical and sub-tropical countries globally^1–7^. About 5.4 million snakebites occur each year, resulting in 1.8 to 2.7 million cases of envenomation with 81,410 to 137,880 deaths and ~400,000 amputations and other permanent disabilities each year^2^. In India alone ~45,900 deaths are recorded annually due to snakebites^3,4^. However, these figures may not be the actual representation as a large number of snakebite envenomation remain unreported^1^. Treatment of snakebite primarily relies upon administration of anti-venom (AV). These AVs are usually obtained from the sera of equines, which have been hyperimmunized with a cocktail of one or more snake venoms and an adjuvant to boost the immune system^7^. Commercial AV producers usually purify the immunoglobulin or F(ab)_2_ fragment from the serum, and in order to improve the stability, it can be lyophilized and transported in the form of dry powder that is reconstituted at the time of use^7^. In absence of an accurate diagnostic test for snakebite, often a large quantity of poly-specific AV is administered in the victim that may increases the risk of various adverse reactions such as urticaria, serum sickness, and life-threatening anaphylaxis due to its poly-specific nature and equine origin^6–10^. In order to avoid such AV-associated complexities, accurate diagnosis of snakebite is very critical^6^ that will allow the administration of a monospecific AV that is reported to have fewer cases of adverse reactions (only 12.9% cases vs 79% cases in poly-specific AV treated subjects)^11,12^. Current diagnostic approaches are centred around manifestations of snakebite associated various symptoms and a blood coagulation test^6^. In recent years, antibody-based immunoassays have gained significant attention in snakebite diagnosis but it suffers from an inherent disadvantage including high-batch-to-batch variation that limit its application in real diagnostic situations^6,13,14^. A further challenge associated with snakebite diagnosis is the variable nature of snake venom, which is a highly complex mixture of proteins, enzymes, carbohydrates, peptides and other substances^1,7,15^ that poses a great challenge for the development of a polyclonal antibody-based specific diagnostic tool. Development of a monoclonal antibody that specifically recognizes a particular venom component is challenging and moreover, maintenance of such clones requires extensive cell culture facilities along with cold storage^6,7,15^. However, the diagnostic challenge posed by venom variability can be addressed by designing a diagnostic reagent with an ability to recognize an epitope shared by venom of geographically distinct population of a snake species.

In recent years, a new class of nucleic acids called ‘aptamers’ have spurred great interest in the scientific community and diagnostic industry owing to their ability to replace antibodies in all possible diagnostic formats^13,16–21^. Aptamers are structured nucleic acids that can be generated through a process of *in vitro* evolution called Systematic Evolution of Ligands by EXponential enrichment (SELEX)^14,19,22,23^. Aptamers are strong chemical rivals of antibodies and are also known as aptabodies or chemical antibodies^22^. They evince several obvious advantages over their antibody counterparts including, but not limited to, high stability, room temperature storage, ease of synthesis and functionalization, ethical advantages as the need for animal subjects is obviated, and negligible batch-to-batch variation. In recent years, aptamer technology has attained an impressive growth in the area of diagnosis, and a large number of aptamers have been reported against various analytes ranging from metal ions, small molecules, protein, toxins and even whole bacterial and cancer cells^14,19,23,24^. More recently, the utility of aptamers has also been demonstrated for the detection of α- bungarotoxin^24^, a major-constituent (~60%) of the venom of *Bungarus multicinctus* (many-branded krait)^7,24^. *Bungarus multicinctus* is a highly venomous snake distributed throughout the South-east Asia and East Asian islands. One of the major constituents of the venom, *α*-Bungarotoxin, binds irreversibly and competitively to the acetylcholine receptor of the neuromuscular junction and induces paralysis, respiratory failure and eventually causes the death of the victim^7^. The Indian counterparts of *B. multicinctus* are *B. caeruleus, B. niger, B. walli, B. sindanus*, and *B. fasciatus*. However, *B. caeruleus* is the most common one and it is an important member of ‘BigFour’ group that cause the majority of envenomation in the Indian subcontinent^25,26^. A BLAST search reveals that the α-bungarotoxin from *B. caeruleus* is ~80% similar to the α-bungarotoxin from *B. multicinctus* **(**Table 1**)**. Based on this information, we hypothesized that an aptamer generated against α-bungarotoxin of *B. multicinctus* should obviate the need for aptamer generation against α-bungarotoxin of *B. caeruleus* (krait) for the diagnosis of krait bite^27^. With this aim, we evaluated the previously reported aptamer (α-Tox-FL) for its ability to detect the venom of *B. caeruleus*. In addition, we showed that the rationally truncated aptamer displayed high affinity and specificity comparable to the parent aptamer. The truncated variant developed in this study may provide a tool for the epidemiological studies and diagnosis of krait bite in Indian context. The study could be a significant contribution to a multi-pronged program to reduce mortality from snakebite in India.

**Table 1:** NCBI BLAST results showing sequence similarity between α-Toxin of *B. multicinctus* and α-Toxin of *B. caeruleus*, *B. fasiatus and B. niger*.

Toxin	Uniprot/NCBI ID	FASTA Sequence	Sequence similarity with*B. caeruleus*
α-Toxin Bungarotoxin *B. multicinctus*	Uniprot P60615 (3L21A_BUNMU)	>sp|P60615|3L21A_BUNMU Alpha-bungarotoxin isoform A31 *Bungarus multicinctus* MKTLLLTLVVVTIVCLDLGYTIVCHTTATSPISAVTCPPGENLCYRKMWCDAFCSSRGKVVELGCAATCPSKKPYEEVTCCSTDKCNPHPKQRPG	80%
α-Toxin *B. caeruleus*	Uniprot D2N116 (3L2A_BUNCE)	>sp|D2N116|3L2A_BUNCE Alpha-delta-bungarotoxin-4 (Fragment) *Bungarus caeruleus* YTLLCHTTSTSPISTVTCPSGENLCYTKMWCDAFCSSRGKVIELGCVATCPQPKPYEEVTCCSTDKCNPHPKQRPG	*B.caeruleus* α-Toxin Sequence used to compare sequence similarity
α-Toxin *B. fasciatus*	Uniprot C0HJI9 (3S11B_BUNFA)	>sp|C0HJI9|3S11B_BUNFA Alpha-elapitoxin-Bf1b (Fragment) *Bungarus fasciatus* RICLNQQSSEPQTTEICPDGEDTCY	27%
α-Toxin *B. niger*	Txid 282406	Cytochrome b> CAG38945.1 cytochrome b *Bungarus niger* MSNQHILLISNLLPVGSNISTWWNFGSMLMTCLLLQIMTGFFLAIHYTANINLAFSSTVHIMRDVPYGWTMQNIHAISASLFFICIYIHIARGLYYGLYLNKEVWLSGTALLITLMATAFFGYVLPWGQMSFWAATVITNLLTAIPYLGNTLTTWLWGGFSINDPTLTRFFALHFILPFAIISLSSIHILLLHNEGSNNPLGTNSDIDKIPLHPYHSYKDMLMITIMITLLFTILSFMPNLLNDPENFSKANPLLTPQHIKPEWYFLFAYGILRSIPNKLGGTMALIMSVAILISAPFTHTSYIRSMAFRPLTQILFWTLVSTFIIITWTATKPVESPFIFISQMTSVIYFSFFIINPLLGWTENKIMMSCPSSLP NADH dehydrogenase subunit 4 > CAH25761.1 NADH dehydrogenase subunit 4, *Bungarus niger* PIAGSMVLAAILLKLGGYGIIRMSQILPLLKTDMFLPFIVLSLWGAILASLTCLQQTDLKSLIAYSSISHMGLVIAAISIQTQWSLVGAMMMMIAHGFTSSALFCLANSTYERTQTRIMILTRGFHNILPMTTSWWLLANLMNIATPPSINFTSELLIASSLFNWCPTSIILFGLLMLITASYSLHMFLSTQMNTMMLNTPIQPALSREHLIMTLHIIPLMLISLKPELVI	6% No sequence similarity

## Materials and Methods

### Reagents and Chemicals

All routine reagents were procured from Sigma Aldrich, USA, unless otherwise mentioned. Oligonucleotides used in the study were procured from Integrated DNA Technologies (IDT, USA). 96 well plates (MaxiSorp^TM^) were procured from Nunc, USA. Bovine Serum Albumin (BSA) and 4-(2-hydroxyethyl)-1-piperazineethanesulfonic acid (HEPES) was procured from Sigma Aldrich, USA. 3, 3′, 5, 5′-tetramethylbenzidine (TMB) (BD OptEIA™) was procured from BD Biosciences, USA. Snake venom was obtained from wild specimens collected under permit numbers 5141/WL/4R-6/2017, A.33011/5/2011-CWLW/305, and WL/Research Study/WLM/2341 issued by West Bengal, Mizoram and Himachal Pradesh Forest Departments, India, which were released after milking and whose specific identity was confirmed by expert herpetologists. Snakes were handled according to relevant guidelines or regulations. Ethical permission to obtain snake venom from wild snake was taken from the Institutional Ethics Committee, Bangor University UK and the Calcutta National Medical College, India. In addition to this, snake venom was also procured from K.V. Institute Uttar Pradesh, India. Premium Serums and Vaccines Pvt. Ltd. Pune, Maharashtra, India has also provided venom of ‘Big Four’ species as a generous gift. Permission to handle the snake venom at THSTI was taken from the Institutional Biosafety Committee, THSTI, Faridabad-121001, Haryana, India.

### Protein BLAST Search

In order to assess the sequence similarity between α-bungarotoxin of *B. multicinctus and B. caeruleus* protein sequence of α-bungarotoxin from both the aforementioned species was subjected to NCBI protein BLAST search (https://blast.ncbi.nlm.nih.gov). Protein sequence of α-bungarotoxin of *B. multicinctus* was used as a query while Protein sequence of α-bungarotoxin of *B. caeruleus* was used as a subject **(**Table 1**)**.

### Truncation of Aptamer

α-bungarotoxin specific 40-mer aptamer (α-Tox-FL) was submitted to M-fold webserver (http://unafold.rna.albany.edu/?q=mfold/DNA-Folding-Form) to predict its secondary structure^28^. Two variants of α-Tox-FL (40-mer) were designed as guided by its secondary structure. These two variants were designated as α-Tox-T1 (14-mer) and α-Tox-T2 (26-mer) which is further assessed for their ability to bind venom of *B. caeruleus* in comparison to its parent aptamer α-Tox-FL.

### Enzyme Linked Aptamer Assay (ELAA)

Binding of α-Tox-FL, α-Tox-T1 and α-Tox-T2 were evaluated using ELAA as described by Ye *et al*.^7^. Briefly, a 96-wellplate was coated with 1 µg/well venom of *B. caeruleus* in 100 µL (0.1 mol/L) sodium bicarbonate buffer pH 9.6, followed by two washes with 200 µL wash buffer (20 mM HEPES pH 7.4, supplemented with 150 mM NaCl, 5 mM KCl, 2 mM MgCl_2_ and 2 mM CaCl_2_) containing 0.05% Tween 20. Wells were then blocked with 100 µL of 1 mg/mL BSA solution in binding buffer (was buffer without Tween-20) for 2 hr at room temperature (RT). After blocking, the wells were washed twice with wash buffer to remove unbound protein. The biotinylated aptamers (50 pmol) were heated at 92 °C for 10 min, cooled on ice and brought to RT. Fifty picomole (per well) of 5′ biotinylated aptamer was then added to the individual wells followed by 40 minutes incubation at RT. Subsequently, the wells were washed 3 times with wash buffer. Following this, 100 µL of 1:1000 dilution of streptavidin-HRP conjugate in binding buffer was added per well for 40 minutes at RT. The wells were washed as previously described. TMB (100 µL) was added as a substrate to each well, incubated for 5 min and the reaction was quenched by adding 100 µL of 5% H_2_SO_4_ to each well. The venom-bound aptamer-streptavidin complex was quantified at 450 nm (O.D._450_) using M2e plate reader (Molecular Devices USA). In this experiment, a buffer-coated well was served as an antigen control and data was plotted as ΔO.D. 450 nm (O.D.450 nm of test - O.D.450 nm of antigen control).

### Cross-reactivity testing

The selected aptamer candidates were tested in ELAA format against 8 different snake venoms including *B. caeruleus*, namely venom obtained from *Nana naja, N. kaouthia, N. oxiana, Daboia russelii*, *B. caeruleus, B. fascinatus, B. niger*, and *Echis carinatus*. These species were chosen based on their distribution and importance in terms of snakebite in the Indian subcontinent. In order to examine the cross reactivity of parent and truncated aptamer candidates, 1 µg/well of each venom was coated on to a 96-well plate in 100 µL of coating buffer and kept at 4 °C overnight. The rest of the ELAA procedure was performed as described in the ELAA section above. A heat map was created as a function of absorbance of each aptamer candidate for tested venoms.

### Circular Dichroism

The Circular Dichroism (CD) experiment was performed on a J-815 Spectropolarimeter (JASCO, Tokyo Japan) to evaluate the secondary structure of full length and truncated aptamer candidates. A quartz cuvette with 0.2 cm path length was used to record the spectra of samples containing 20 μM of each aptamer (α-Tox-FL, α-Tox-T1 and α-Tox-T2) in binding buffer.

### Determination of apparent dissociation constant (K_d_) of aptamer candidates

To determine the K_d_ of parent and the truncated aptamer candidate against venom of *B. caeruleus*, 1 µg venom of *B. caeruleus*/well was coated at 4 °C overnight on a 96 well plate in 100 µL of coating buffer. After blocking with 100 µL of 1 mg/mL BSA solution prepared in binding buffer, a range of aptamer concentrations (2–500 nM) was added. ELAA was performed as described above. O.D._450_ was plotted as a function of aptamer concentration and K_d_ was determined by the below mentioned non-linear regression for one-site binding using Graph-pad Prism version 5.02. Y represents the aptamer binding, X is aptamer concentration and Bmax is maximum binding.

$${\bf{Y}}={\bf{Bmax}}\times {\bf{X}}/({\bf{Kd}}+{\bf{X}})$$

### Limit of detection of parent and truncated aptamer

The limit of detection (LOD) of venom of *B. caeruleus* for parent and truncated variant was determined by ELAA. In each case, a range of *B. caeruleus* venom was taken (2–1000 ng/well). Rest of the procedure remain the same as followed for ELAA.

### Evaluation of aptamer binding for geographically distinct venoms

In order to demonstrate the application of aptamer for species identification, we have evaluated the aptamer binding against venom using ELAA as described above. For this, the geographically distinct population of *Bungarus caeruleus* and other species from two different states of India (Uttar Pradesh and Maharashtra) was used in this assay.

## Results and Discussion

The present work establishes the role of truncation in designing shorter yet effective aptamer for the detection of venom of *B. caeruleus*. This was achieved through sequential approaches starting with the BLAST search of α-bungarotoxin of *B. multicinctus* and *B. caeruleus*, followed by secondary-structure guided truncation of α-Tox-FL.

### Protein BLAST Search

Protein BLAST Search of the protein sequence of α-bungarotoxin of *B. multicinctus* and *B. caeruleus* revealed that these toxins share ~87% identity with 80% query coverage **(**Table 1**)** despite the species-level differences. However, it shows poor sequence similarity with *B. fasciatus and B. niger*. Based on this observation, we hypothesized that the aptamer generated for α-bungarotoxin of *B. multicinctus* should have high probability of binding to venom of *B. caeruleus* that also contains α-bungarotoxin, with minor differences in comparison to α-bungarotoxin of *B. multicinctus*.

### Truncation of *B. multicinctus* α-Toxin specific aptamer

**Figure 1:**
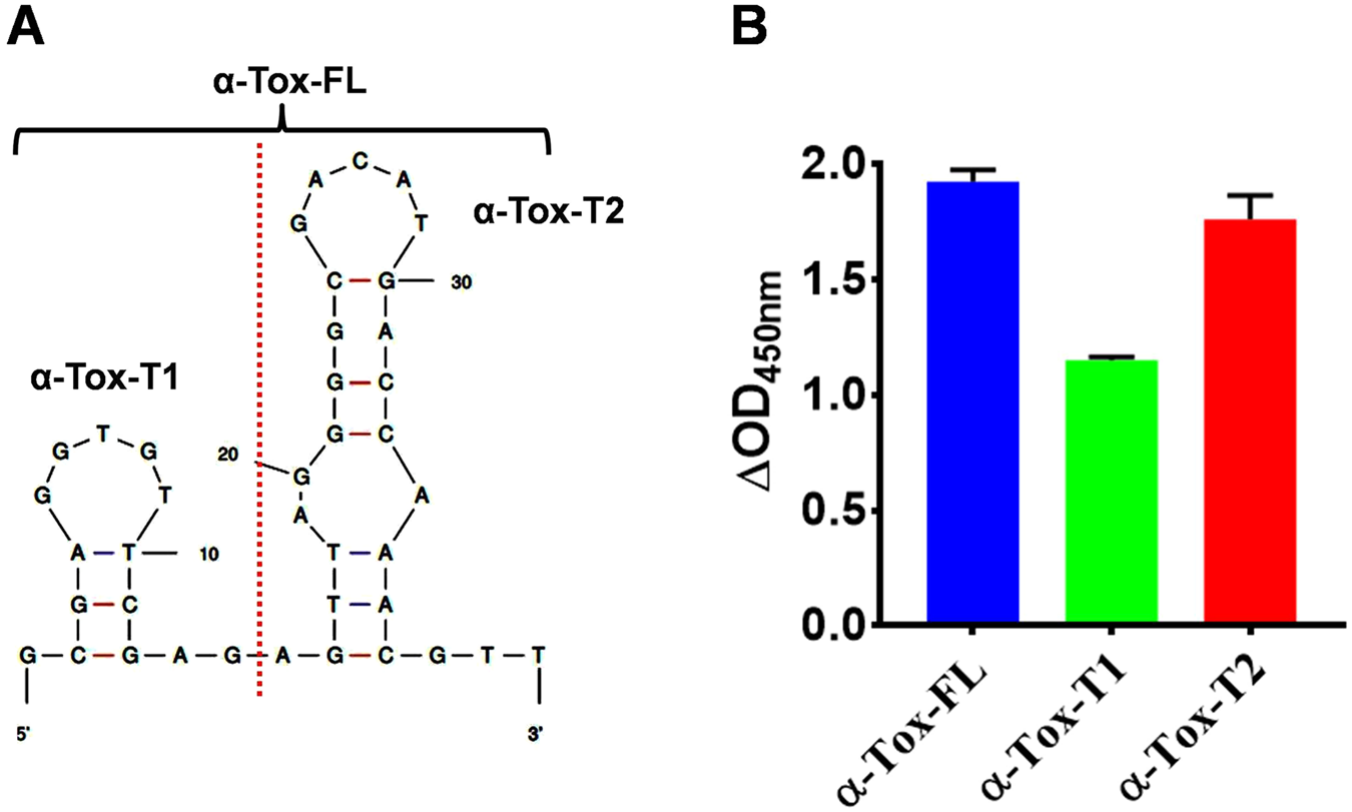
**(A**) Secondary structure of α-Tox-FL, α-Tox-T1 and α-Tox-T2 aptamers as obtained by Mfold. Red dotted line represents point of truncation. **(B)** Showing a comparison of binding of α-Tox-FL, α-Tox-T1 and α-Tox-T2 aptamers to *B. caeruleus* venom by ELAA.

As it is well known fact that only a portion of aptamer is critical for its function and target binding and truncation of aptamer may leads to improvement in aptamer binding^14,16,29^. Thus, as a first step to establish the structure-activity relationship α-Tox-FL (40-mer DNA aptamer) developed for α-Toxin of *B. multicinctus*, it was first subjected to Mfold analysis using DNA parameters^28^. Mfold predicted a secondary structure of α-Tox-FL with a minimum free energy of −4.06 kcal/mol. This secondary structure predicted by Mfold **(**Fig. 1A**)** evinced that the α-Tox-FL contain two stem-loop-like structures. Therefore, to establish their independent role we have truncated the parent aptamer into two secondary structures guided truncated variant (α-Tox-T1 and α-Tox-T2). The first variant (α-Tox-T1) is a 14-mer stem-loop-like structure having three Watson-Crick base-pairs (AT and two GC repeats) in the stem while the loop is formed by 5′ GGTGT 3′ sequence. The secondary structure of α-Tox-T2 is relatively complex and it contains two stems. Stem-1 contain three Watson-Crick base-pairs (AT and two GC repeats, similar to the stem of α-Tox-T1) while Stem-2 contains three Watson-Crick base-pairs (3GC repeats) and a non-Watson-Crick base-pair (GA). Loop-1 that connects Stem-1 and Stem-2 is formed by a 5′ AGGCA 3′ sequence while Loop-2 associates with Stem-2 of α-Tox-T2 and formed by 5′ GACAT 3′. To evaluate the effect of truncation on binding to α-bungarotoxin of *B. caeruleus*, all three 5′ biotinylated aptamer (α-Tox-FL, α-Tox-T1 and α-Tox-T2) was assessed through ELAA. ELAA data clearly demonstrated that all three aptamer candidates can bind to the venom of *B. caeruleus* **(**Fig. 1B**)**. However, it is evident that binding of α-Tox-T2 is comparable to the binding of the parent aptamer α-Tox-FL, while α-Tox-T1 play only minor role in binding **(**Fig. 1B**)**. This result unequivocally demonstrates the power of post-SELEX optimization and rational engineering of an aptamer to identify the smallest functional unit within the aptamer. These findings are in agreement with the previous reports suggesting that the truncated aptamer can perform comparable or sometimes superior to the parent aptamer candidate^14,29,30^.

### Cross-reactivity testing

**Figure 2:**
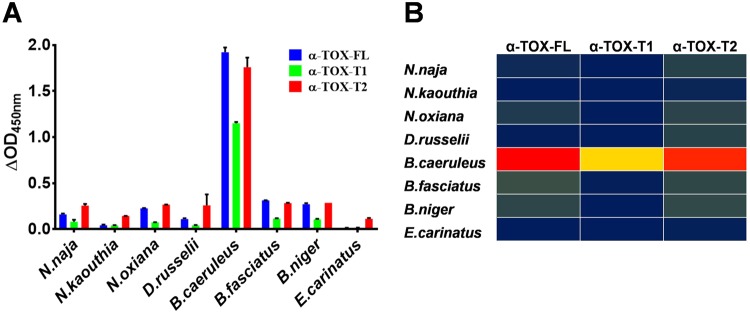
**(A**) Showing ELAA results representing selectivity of α-Tox-FL, α-Tox-T1 and α-Tox-T2 aptamers to *B. caeruleus* venom. **(B**) Selectivity of α-Tox-FL, α-Tox-T1 and α-Tox-T2 aptamers to *B. caeruleus* venom is represented as three-colour gradient heat map (a heat-map representation of ELAA response). Red colour indicates highest binding while blue represent the lowest binding.

The parent aptamer (α-Tox-FL) was identified for α-bungarotoxin of *B. multicinctus* by Veedu’s group^24^ but the potential of this aptamer to detect this toxin in crude venom or its specificity, was not established. Here, the ELAA data (Fig. 2A) unequivocally demonstrated that all three aptamers have very high selectivity for venom of *B. caeruleus* in comparison to the venom of other seven species tested. The binding of individual aptamer candidates to the tested venom is shown in the form of a three-colour gradient heat-map (red coloured box depicts the highest binding while blue represent the lowest binding) **(**Fig. 2B**)**. Taken together, binding and selectivity of α-Tox-T2 is comparable to its parent counterpart. Although α-Tox-T1 retains high selectivity, its binding is poor in comparison to α-Tox-FL and α-Tox-T2. Interestingly, despite the fact that when α-Tox-FL was identified, no crude venom was used to rule out the cross-reactivity^24^ of the aptamer pool against venom of the species used in the current study but none of the aptamer has shown cross-reactivity with the venom of other species. Conclusively, aptamer designed for α-Toxin of *B. multicinctus* has been shown to efficiently detect α-Toxin of *B. caeruleus* in crude venom, owing to the primary sequence similarity (~80%) between α-Toxins derived from these two species **(**Table 1**)**. However, it was unable to detect α-Toxin from other *Bungarus sps* (*B. fasciatus and B. niger)*. A plausible explanation could be that α-Toxins of *B. fasciatus and B. niger* has lower sequence similarity (26% and 6% respectively).

### Circular Dichroism (CD)

**Figure 3:**
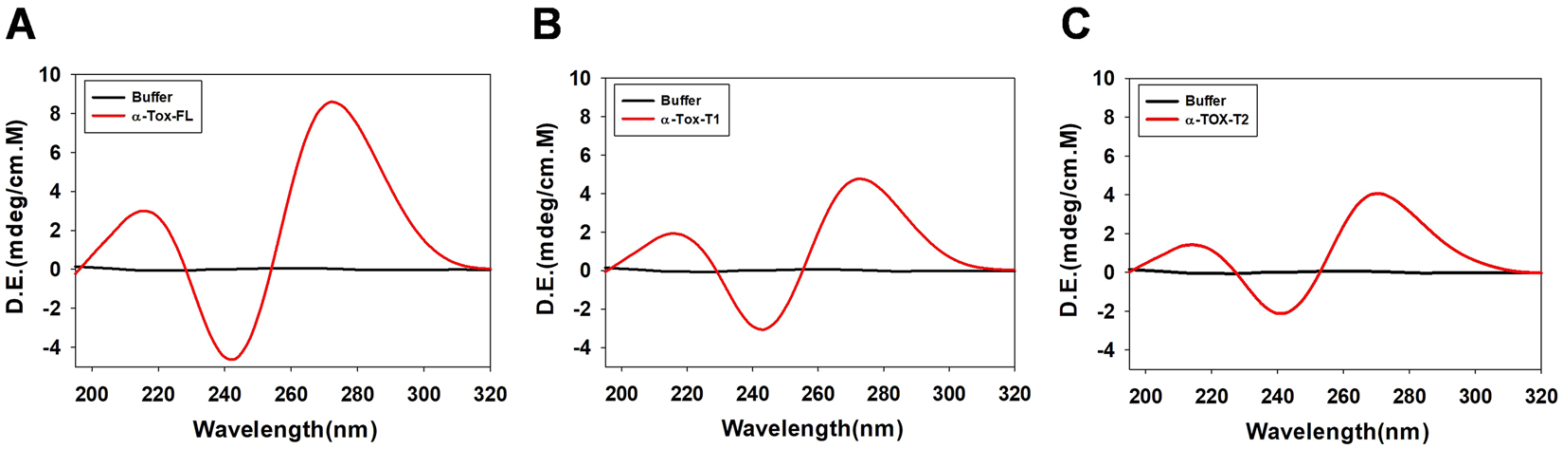
Circular dichroism (CD) spectrum of α-Tox-FL **(A)**, α-Tox-T1 **(B)** and α-Tox-T2 **(C)** aptamers. CD spectra indicate typical B-type stem-loop structure of aptamer that remain unaltered even after truncation.

Next, the change in the structure of the aptamers, if any, as a result of truncation was studied using CD. The CD spectrum of α-Tox-FL displayed a negative peak at 246 nm and two positive peaks at 218 nm and 272 nm, signifying a stem loop type B-DNA structure^14,31^ **(**Fig. 3A**)**. Similarly, α-Tox-T1 and α-Tox-T2 showed negative peak at 246 nm and two positive peaks at 218 nm and 272 nm confirming the formation of a stem loop type B-DNA structure similar to the parent **(**Fig. 3B,C**)**. It is evident from the CD data that the secondary structure of the truncated aptamers remains unaltered.

### Apparent dissociation constant (K_d_) of aptamer candidates

**Figure 4:**
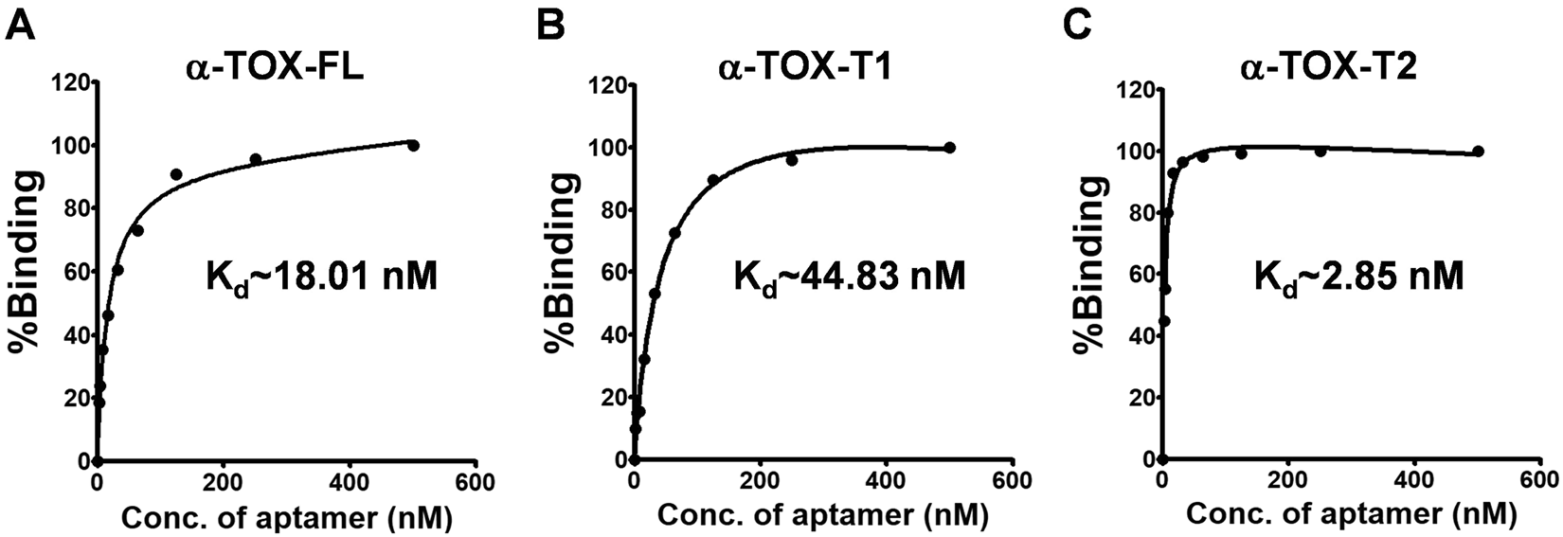
Apparent dissociation constant curve derived through non-linear regression representing binding affinity (K_d_) of α-Tox-FL **(A**), α-Tox-T1 **(B)** and α-Tox-T2 **(C)** aptamers.

The apparent dissociation constants (K_d_) of α-Tox-FL, α-Tox-T1 and α-Tox-T2 aptamers were determined by non-linear regression method for one-site binding (Graph-pad Prism version 5.02). The aptamers were ranked in terms of K_d_ (highest to lowest affinity) as α-Tox-T2 (2.85 nM) > α-Tox-FL (18.01 nM) > α-Tox-T1 (44.83 nM, Fig. 4). α-Tox-T2 has ~6.3 fold higher affinity than its parent aptamer, suggesting that truncation leads to improved affinity in case of α-Tox-T2. On the other hand, α-Tox-T1 display poor affinity suggesting that alone it does not have any significant role in binding. These results are in agreement with the previous reports demonstrating that truncation leads to an improvement in affinity^14,29,30^.

### Limit of detection of parent and truncated aptamer

**Figure 5:**
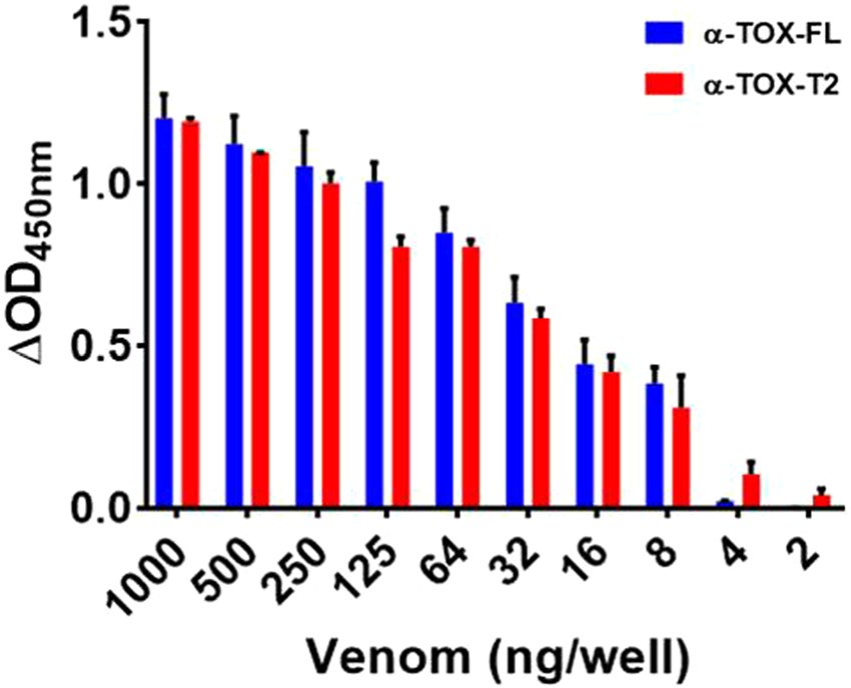
Low end detection limit of α-Tox-FL and α-Tox-T2 aptamers. α-Tox-T2 represents more sensitive detection of *B. caeruleus* venom in comparison to its parent counterpart.

The truncated aptamer α-Tox-T2 could detect *B. caeruleus* venom in as low quantities as 2 ng crude venom, while the parent aptamer α-Tox-FL has a lower detection limit of 8 ng venom under a similar set of conditions **(**Fig. 5**)**. This finding clearly demonstrates that, in terms of lower-end detection limit, α-Tox-T2 is superior to its parent aptamer. The possible reason for the superior detection by the truncated aptamer is due to its improved affinity over parent aptamer.

### Evaluation of aptamer binding for geographically distinct venoms

**Figure 6:**
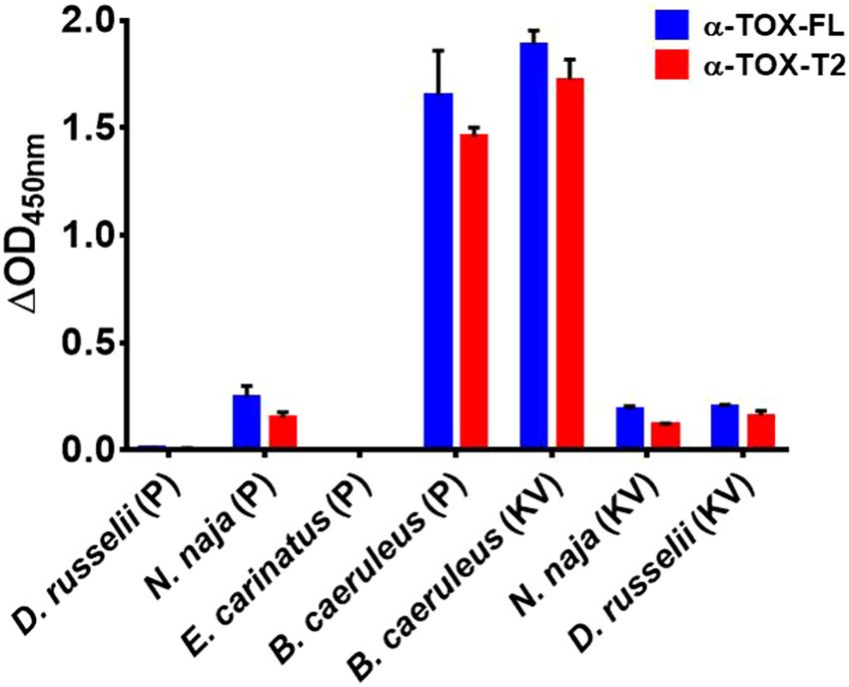
Binding of aptamer to geographically distinct venom of *B. caeruleus* and other members of ‘BIG Four’ group. KV in parentheses represents venom procured from KV Institute Uttar Pradesh India while P in parentheses represent venom obtained from Premium Serums and Vaccines Pvt.Ltd. Pune, Maharashtra, India.

To demonstrate the application of aptamer for species identification, in addition to the snake venom obtained from West Bengal (eastern part of India, Figs 1 and 2) we have also evaluated the aptamer binding against venom obtained from geographically distinct populations of Bungarus caeruleus and other species from two other states, Uttar Pradesh and Maharashtra (northern and western regions of India respectively). ELAA results **(**Fig. 6**)** clearly demonstrated that both the parent and truncated aptamer selectively binds to the venom of Bungarus caeruleus regardless of geographical origin. This result clearly indicates that aptamer is possibly binding to an aptatope which is common in the venoms of Bungarus caeruleus used in the current study.

## Conclusion

As snake venom is a highly complex mixture of various biological molecules ranging from carbohydrates, protein, enzymes, lipids and other substances, specific detection of snake venom is highly challenging and often cannot be achieved by using antibodies, as they show high cross-reactivity. Thus, to address this challenge as a proof-of-concept study, we have shown here that rational truncation is an efficient strategy to develop shorter yet efficient binders from the parent aptamer without compromising its affinity, selectivity and secondary structure. Importantly, we have demonstrated that if the aptamer and target selection is made intelligently then the aptamer can have cross-species application as shown in the current study, in which an aptamer designed for α-Toxin of *B. multicinctus* has been shown to efficiently detect α-Toxin of *B. caeruleus* in crude venom. Being a synthetic reagent, aptamers obviate the need for the resource-intensive facilities required for antibody production. Further, there is enormous potential in the diagnostic application of aptamers for the other components present in venom of other snake species. To take this proof of concept to the next level of evaluation, a decent number of krait bites and control samples derived from geographically distinct patients of different age groups is required in future. Such evaluation will certainly pave the way for translation of the aptamer-based diagnostic test for point-of-care diagnosis of Indian krait bite.

## References

[1] Gupta YK, Peshin SS (2014). Snake Bite in India: Current Scenario of an Old Problem. Journal of Clinical Toxicology.

[2] Snakebite envenoming-World Health Organization http://www.who.int/en/news-room/fact-sheets/detail/snakebite-envenoming.

[3] Menon JC, Joseph JK, Whitaker RE (2017). Venomous Snake Bite in India - Why do 50,000 Indians Die Every Year?. J Assoc Physicians India.

[4] Mohapatra B (2011). Snakebite mortality in India: a nationally representative mortality survey. PLoS neglected tropical diseases.

[5] Shaikh IK, Dixit PP, Pawade BS, Waykar IG (2017). Development of dot-ELISA for the detection of venoms of major Indian venomous snakes. Toxicon: official journal of the International Society on Toxinology.

[6] Theakston RD, Laing GD (2014). Diagnosis of snakebite and the importance of immunological tests in venom research. Toxins.

[7] Ye F (2014). Recognition of Bungarus multicinctus venom by a DNA aptamer against beta-bungarotoxin. PloS one.

[8] de Silva HA, Ryan NM, de Silva HJ (2016). Adverse reactions to snake antivenom, and their prevention and treatment. British journal of clinical pharmacology.

[9] Morais, V. M. & Massaldi, H. Snake antivenoms: adverse reactions and production technology. *J. Venom. Anim. Toxins incl. Trop. Dis* **15** (2009).

[10] Ryan NM, Kearney RT, Brown SG, Isbister GK (2016). Incidence of serum sickness after the administration of Australian snake antivenom (ASP-22). Clinical toxicology.

[11] Amin, M. R. *et al*. Anti-snake venom: use and adverse reaction in a snake bite study clinic in Bangladesh. *J. Venom. Anim. Toxins incl. Trop. Dis* **14** (2008).

[12] Poovazhagi, V. *et al*. Anti-snake venom induced reactions among children with snake envenomation. *International Journal of Contemporary Pediatrics* **4** (2017).

[13] Dhiman A, Kalra P, Bansal V, Bruno JG, Sharma TK (2017). Aptamer-based point-of-care diagnostic platforms. Sensors and Actuators B: Chemical.

[14] Sharma TK, Bruno JG, Dhiman A (2017). ABCs of DNA aptamer and related assay development. Biotechnol Adv.

[15] Gutierrez, J. M. *et al*. Preclinical Evaluation of the Efficacy of Antivenoms for Snakebite Envenoming: State-of-the-Art and Challenges Ahead. *Toxins* **9**, 10.3390/toxins9050163 (2017). 10.3390/toxins9050163 PMC5450711 28505100

[16] Generation and application of DNA aptamers against HspX for accurate diagnosis of tuberculous meningitis Tuberculosis **112**, 27–36 (2018). 10.1016/j.tube.2018.07.004 30205966

[17] Clinical utility of aptamer-based point-of-care antigen detection in sputum for the diagnosis of pulmonary tuberculosis. ACS Infectious Disease (Manuscript ID-2018-00201k).

[18] A novel aptamer-based test for the rapid and accurate diagnosis of pleural tuberculosis. 10.1016/j.ab.2018.10.019 30352198

[19] Sharma TK (2014). Aptamer-mediated ‘turn-off/turn-on’ nanozyme activity of gold nanoparticles for kanamycin detection. Chemical communications.

[20] Sharma TK, Bruno JG, Cho WC (2016). The Point behind Translation of Aptamers for Point of CareDiagnostics. Aptamers and Synthetic Antibodies.

[21] Harleen Kaur H, Bruno JG, Kumar A, Sharma TK (2018). Aptamers in the Therapeutics and Diagnostics Pipelines. Theranostics.

[22] Kalra P, Dhiman A, Cho WC, Bruno JG, Sharma TK (2018). Simple Methods and Rational Design for Enhancing Aptamer Sensitivity and Specificity. Frontiers in molecular biosciences.

[23] Chopra A, Shukla R, Sharma TK (2014). Aptamers as an emerging player in biology. Aptamer and Synthetic Antibodies.

[24] Lauridsen LH, Shamaileh HA, Edwards SL, Taran E, Veedu RN (2012). Rapid one-step selection method for generating nucleic acid aptamers: development of a DNA aptamer against alpha-bungarotoxin. PloS one.

[25] Hiremath V (2016). Differential action of medically important Indian BIG FOUR snake venoms on rodent blood coagulation. Toxicon: official journal of the International Society on Toxinology.

[26] Choudhury M, McCleary RJR, Kesherwani M, Kini RM, Velmurugan D (2017). Comparison of proteomic profiles of the venoms of two of the ‘Big Four’ snakes of India, the Indian cobra (Naja naja) and the common krait (Bungarus caeruleus), and analyses of their toxins. Toxicon: official journal of the International Society on Toxinology.

[27] Vinod KV, Dutta TK (2013). Snakebite, dysautonomia and central nervous system signs. QJM: monthly journal of the Association of Physicians.

[28] Zuker M (2003). Mfold web server for nucleic acid folding and hybridization prediction. Nucleic acids research.

[29] Macdonald J, Houghton P, Xiang D, Duan W, Shigdar S (2016). Truncation and Mutation of a Transferrin Receptor Aptamer Enhances Binding Affinity. Nucleic acid therapeutics.

[30] Kaur H, Yung LY (2012). Probing high affinity sequences of DNA aptamer against VEGF165. PloS one.

[31] Kypr J, Kejnovska I, Renciuk D, Vorlickova M (2009). Circular dichroism and conformational polymorphism of DNA. Nucleic acids research.

